# Magnetism beyond the Monolayer: Long-Range Order in an Interfacial Phase of CoBr_2_

**DOI:** 10.1021/acs.nanolett.6c03267

**Published:** 2026-10-19

**Authors:** Samuel Kerschbaumer, David Serrate, Adriana Elizabet Candia, Andrea Aguirre, Danilo Longo, Fadi Choueikani, Jorge Lobo-Checa, Pavel Jelínek, Maxim Ilyn, Celia Rogero, Sebastien E. Hadjadj

**Affiliations:** † 16379 Centro de Física de Materiales (CSIC/UPV-EHU), 20018 Donostia-San Sebastian, Spain; ‡ Christine Technologies Inc., Sacramento, California 95811, United States; ¶ Instituto de Nanociencia y Materiales de Aragón (INMA), CSIC-Universidad de Zaragoza, 50009 Zaragoza, Spain; § Departamento de Física de la Materia Condensada, Universidad de Zaragoza, 50009 Zaragoza, Spain; ∥ Laboratorio de Microscopias Avanzadas (LMA), Universidad de Zaragoza, 50018 Zaragoza, Spain; ⊥ Instituto de Física del Litoral, Consejo Nacional de Investigaciones Cientificas y Tecnicas, Universidad Nacional del Litoral (IFIS-Litoral, CONICET-UNL), 3000 Santa Fe, Argentina; # 138823 CIC nanoGUNE-BRTA, 20018 Donostia-San Sebastian, Spain; @ Synchrotron SOLEIL, 91190 Saint-Aubin, France; △ FZU - Institute of Physics of the Czech Academy of Sciences, Cukrovarnická 10, 162 00 Prague 6, Czech Republic

**Keywords:** 2D ferromagnetic materials, van der Waals semiconductors, spin-polarized tunneling microscopy and spectroscopy, epitaxial growth of ultrathin films, transition metal dihalides

## Abstract

Transition metal dihalides are promising magnetic van der Waals materials because of their structural simplicity, chemical stability, and compatibility with nanofabrication. While magnetic order in these systems is typically associated with bulk-like monolayers, its relation to interfacial phases remains largely unexplored. Here we show that in CoBr_2_ grown on Au(111), long-range magnetic order already emerges in a structurally distinct interfacial precursor phase, prior to the formation of the bulk-like monolayer. During growth, this phase evolves into the monolayer, accompanied by a transition from molecular-like states to semiconducting behavior. Spin-resolved STM reveals an inhomogeneous in-plane magnetic response that is locally suppressed by point defects, whereas macroscopic XMCD demonstrates robust long-range order with pronounced in-plane anisotropy. These results establish that long-range magnetic order in CoBr_2_ does not require an ideal bulk-like monolayer but can emerge from interfacial phases with distinct electronic and structural properties, enabling magnetism beyond the monolayer limit.

## Graphical abstract

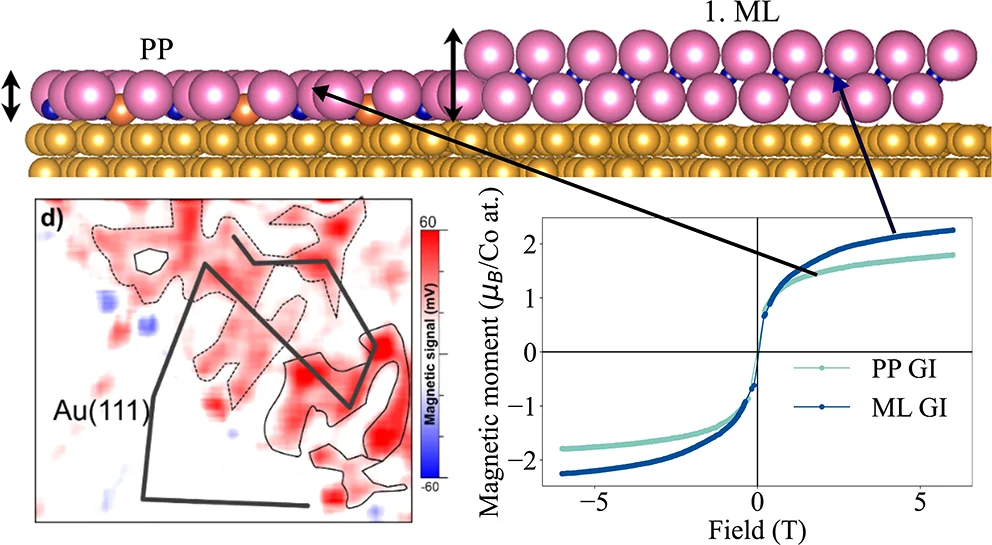

The rapid development of intrinsically magnetic two-dimensional (2D) materials has opened new directions in condensed-matter physics, where reduced dimensionality, spin–orbit coupling, and interfacial engineering combine to produce emergent quantum phenomena absent in bulk magnets.^1–6^ The discovery of long-range magnetic order in van der Waals crystals such as CrI_3_ and Cr_2_Ge_2_Te_6_ demonstrated that magnetic anisotropy can stabilize magnetism in the 2D limit.^7,8^ Since then, the family of 2D magnets has expanded to include metallic Fe_3_GeTe_2_ and semiconducting or insulating systems such as MnBi_2_Te_4_ and transition metal dihalides (TMDHs), where competing exchange interactions and anisotropies give rise to a wide range of magnetic ground states.^9–13^

Understanding how structural and electronic properties evolve at the atomic-scale interface, and how these changes affect magnetic order, is therefore essential for controlling magnetic behavior in low-dimensional systems and for designing functional magnetic interfaces. Within this framework, TMDHs have emerged as model systems for exploring magnetism in the 2D limit. When grown on metallic substrates, their interfacial geometry can deviate substantially from the bulk structure, profoundly influencing electronic hybridization and magnetic anisotropy.^11,13–15^

Recent studies have shown that distinct interfacial structural phases can form during the early growth stages of FeBr_2_ and CoBr_2_ on Au(111).^11,15^ While magnetic order has been reported in related bromide systems, such as FeBr_2_, its microscopic origin and its relation to interfacial structure and electronic properties remain largely unexplored.

In this work, we focus on the CoBr_2_/Au­(111) system to directly address this question. Upon deposition, CoBr_2_ forms an interfacial precursor phase (PP) that evolves into a bulk-like monolayer (ML) as the coverage increases.^15^ Because these two phases have distinct atomic arrangements, comparing them allows us to directly observe how structural changes influence the material’s electronic and magnetic properties. By combining atomic-scale (STM and spin-polarized STM) and macroscopic (XAS and XMCD) measurements, we establish a direct correlation between local electronic coherence and collective magnetic behavior, demonstrating that long-range magnetic order can already emerge in the interfacial phase.

In order to investigate this relationship, our focus is on the structural and magnetic properties of CoBr_2_ on Au(111). The growth of CoBr_2_ on Au(111) proceeds through two distinct structural phases stabilized by the interaction with the substrate.^15^ At submonolayer coverage (Figure 1a), CoBr_2_ forms a surface-stabilized precursor phase (PP) consisting of ordered molecular clusters arranged in a hexagonal network commensurate with the Au(111) lattice. In this phase, Co atoms directly coordinate to the substrate, with additional Au adatoms stabilizing the structure. Upon increasing the coverage (Figure 1b), the PP reorganizes into the octahedral 1T monolayer (ML), where Co atoms are octahedrally coordinated by Br atoms, forming a van der Waals layer (Figure 1c). This transition thus defines a crossover from a substrate-stabilized molecular network to a bulk-like monolayer, providing the structural framework for understanding the electronic and magnetic properties discussed below.

**Figure 1:**
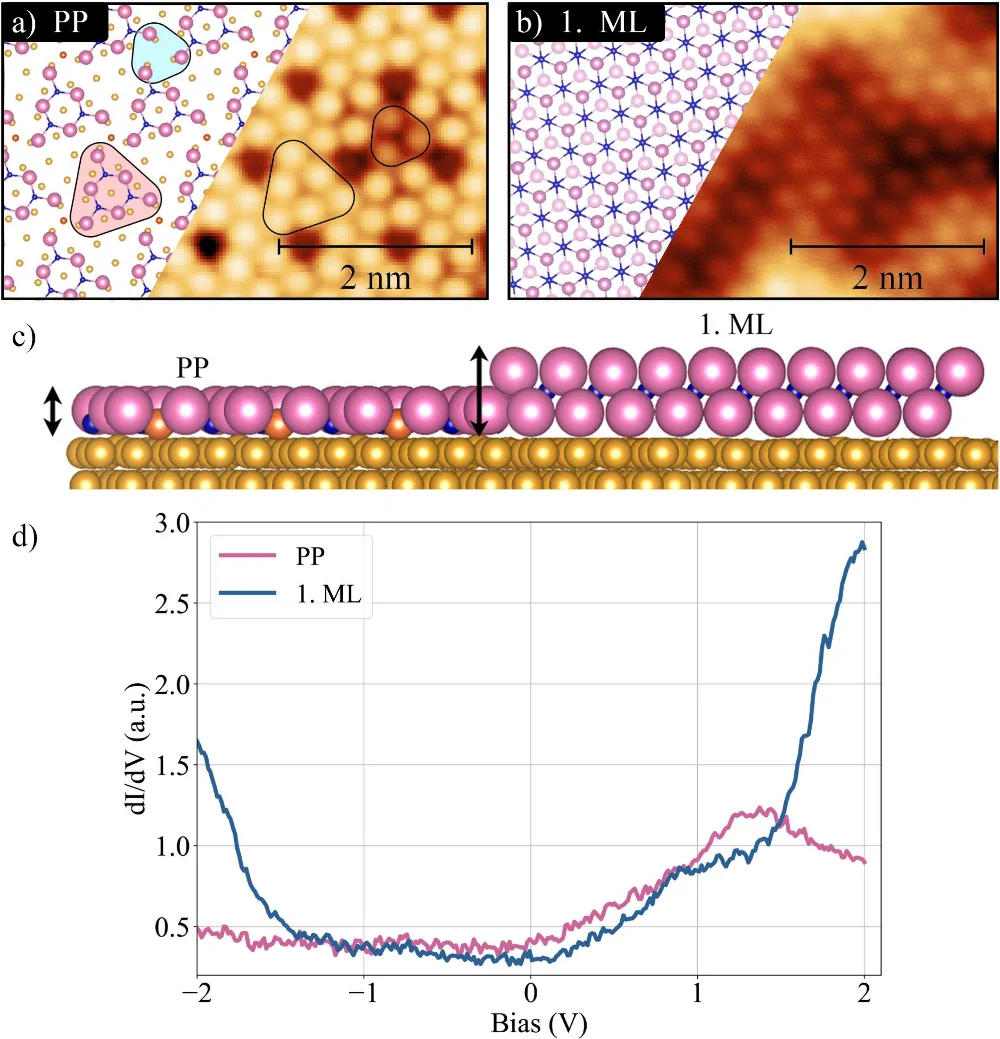
(a, b) Atomic-resolution STM measurements of (a) the PP and (b) 1 ML of CoBr_2_ on Au(111). On the left side of both panels, a top view of the schematic representation of the atomic structure is shown. (c) Lateral representation of the PP and ML phase on Au(111). Color code of the atomic models: Br (pink), Co (blue), Au substrate (gold), Au adatoms (orange). (d) STS measurements of both phases measured at *U* = 0.6 V and *I*_t_ = 30 pA. A direct comparison of the PP spectra with bare Au(111) is given in Figure S8.

The electronic properties of CoBr_2_/Au­(111) (Figure 1d) were investigated by scanning tunneling spectroscopy (STS) on both the precursor phase (PP) and the bulk-like monolayer (ML). Within the accessible bias range, the PP shows no clear onset of valence or conduction bands. At negative bias, the spectra closely resemble those of Au(111), indicating the absence of occupied CoBr_2_-derived states in this energy range. The PP spectra have been characterized in detail with a direct reference to bare Au(111) (Figure S8). However, a bare Au(111) reference cannot, however, be recorded on the sample shown in Figure 1, where PP and ML coexist. First CoBr_2_ forms the PP, which covers the entire Au(111) surface. Only once this coverage is complete does further deposition locally convert the PP into the 1T ML.^15^ For this reason, the PP spectrum serves as the reference for the ML in Figure 1d.

At positive bias, a well-defined, nondispersive resonance appears at +1.3 V. This feature is observed independently of the measurement position, including atomic sites, reconstruction regions, and areas around defects associated with missing Au adatoms,^15^ indicating that it is an intrinsic electronic state of the PP. Notably, this resonance dominates the tunneling conductance and later it will be shown that it coincides with the bias at which spin-polarized STM reveals a clear magnetic contrast, directly linking this state to the magnetic order of the precursor phase.

At higher bias (up to +3 V, Figure S1), an additional localized feature appears at defect sites (black triangles in Figure 1 a). This resonance is therefore assigned to a defect-induced state within the unoccupied energy range.

In contrast to the precursor phase, the bulk-like ML exhibits a well-defined semiconducting gap of approximately 3 eV (see Figure S2), in good agreement with theoretical predictions for the 1T-CoBr_2_ structure. The d*I*/d*V* spectra display clear onsets for both the valence and conduction bands, consistent with the formation of extended electronic states in a two-dimensional lattice. This behavior differs markedly from that of the PP, where no clear valence-band onset is observed and the electronic structure is instead dominated by a localized resonance at +1.3 eV. The sharp, nondispersive nature of this feature indicates a molecular-like state.

At sample biases above +1.0 V, repeated d*I*/d*V* measurements at the same spatial location reveal abrupt changes in the local density of states (Figure 2). These transitions are not accompanied by any detectable topographic modification, indicating that the atomic lattice exposed to the vacuum remains structurally intact. Instead, they reflect a bias-induced instability of the local electronic environment, possibly associated with charge localization, polaron formation, or subtle lattice relaxations beyond the experimental resolution.^14,16,17^ These instabilities hinder reproducible large-range spectroscopic measurements and complicate a direct determination of the band gap. However, their frequency decreases upon repeated high-bias measurements at the same location, eventually leading to stable spectra up to ±2 V.

**Figure 2:**
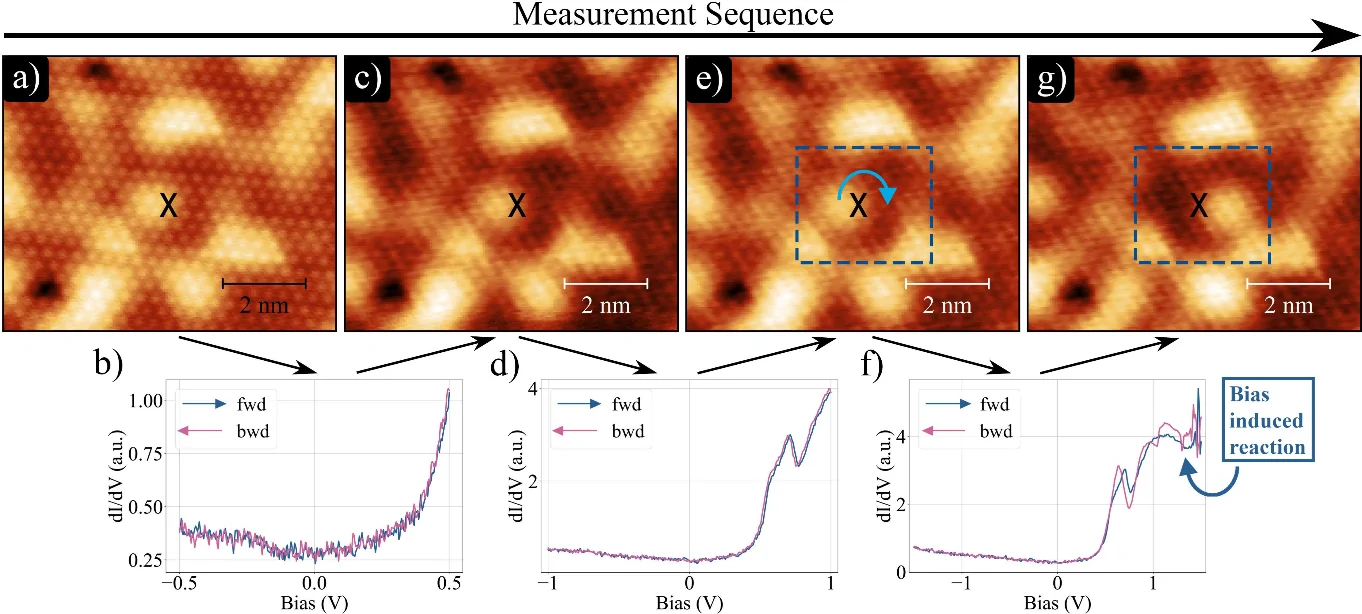
(a, c, e, g) Sequence of STM images of the 1 ML with STS spectra recorded between successive scans at the same position (marked with “X”). (b, d, f) The measured bias range is increased in every subsequently recorded spectrum: (b) ±0.5 V, (d) ±1.0 V, (f) ±1.5 V. We demonstrate that the d*I*/d*V* signal becomes unstable above a critical bias, typically found upon exceeding 1 V (depending on tip conditions, tunneling current or specific location on the surface). This is evident in the last STS measurement, where a discrepancy between the forward (fwd) and backward (bwd) curves is observed. The corresponding STM image reveals a local modification of the electronic surface structure without affecting the atomic positions (blue rectangle). The blue arrow in (e) marks the position where the electronic corrugation changes most after the high-bias STS measurement. The black surface defects serve as fixed landmarks showing that the same area is imaged in all panels. Panel (a) was recorded at a lower scan speed than in (c), (e), and (g) to resolve the atomic lattice. No postprocessing was applied to any of the images. STM: *U* = 500 mV, *I*_t_ = 150 pA. STS: *U* = 500 mV, *I*_t_ = 150 pA, *V*_mod_ = 10 mV.

The sequence in Figure 2c,e,g was recorded as a single continuous series without changing any parameter other than the bias range of the interleaved STS measurement at the position marked “X”. The STM images were acquired at low, nonperturbing bias and are shown without any postprocessing, panel a) was recorded at a lower scan speed to resolve the atomic lattice, which is why atomic resolution is not visible in the faster scans (Figure 2c,e,g). The black defects in the corners of the images are structural surface defects and serve as fixed landmarks, confirming that the same area is imaged throughout the series without lateral drift. Relative to these landmarks, the bright electronic corrugation remains unchanged between panels (c) and (e) and only changes locally around the spectroscopy position between panels (e) and (g) after the STS measurement exceeds +1 V. A change of the tip apex would be expected to alter the contrast of the entire image rather than a single localized region, which leads us to assign this effect to a bias-induced modification of the local electronic structure of the ML. This behavior was observed repeatedly in independent measurement series and sample preparations, always with the same characteristics, and is discussed in more detail in ref 18.

The spectroscopic results provide the basis for probing magnetic order at the atomic scale by defining the relevant energy windows for spin-polarized tunneling measurements. In the bulk-like ML phase, however, the bias-induced instabilities hinder reproducible spectroscopic measurements over a broad energy range, preventing a reliable investigation of spin-dependent contrast.

The precursor phase (PP), in contrast, remains electronically stable under tunneling conditions and exhibits a well-defined localized resonance at +1.3 V. This feature, which dominates the tunneling conductance, provides a suitable energy window to explore magnetic contrast using spin-polarized STM (SP-STM), allowing access to the local magnetic order in this interfacial phase, which is directly relevant for the control of spin-dependent functionalities in low-dimensional systems.^19–23^

Figure 3a,b shows constant-current d*I*/d*V* maps acquired at 1.3 V under high (−2.9 T) and low (0.1 T) magnetic fields on a PP island (Figure 3c). A clear contrast variation is observed between the two field conditions, reflecting a change in the spin-dependent tunneling conductance. The corresponding difference image (Figure 3d) directly maps the local spin density of the precursor phase.

**Figure 3:**
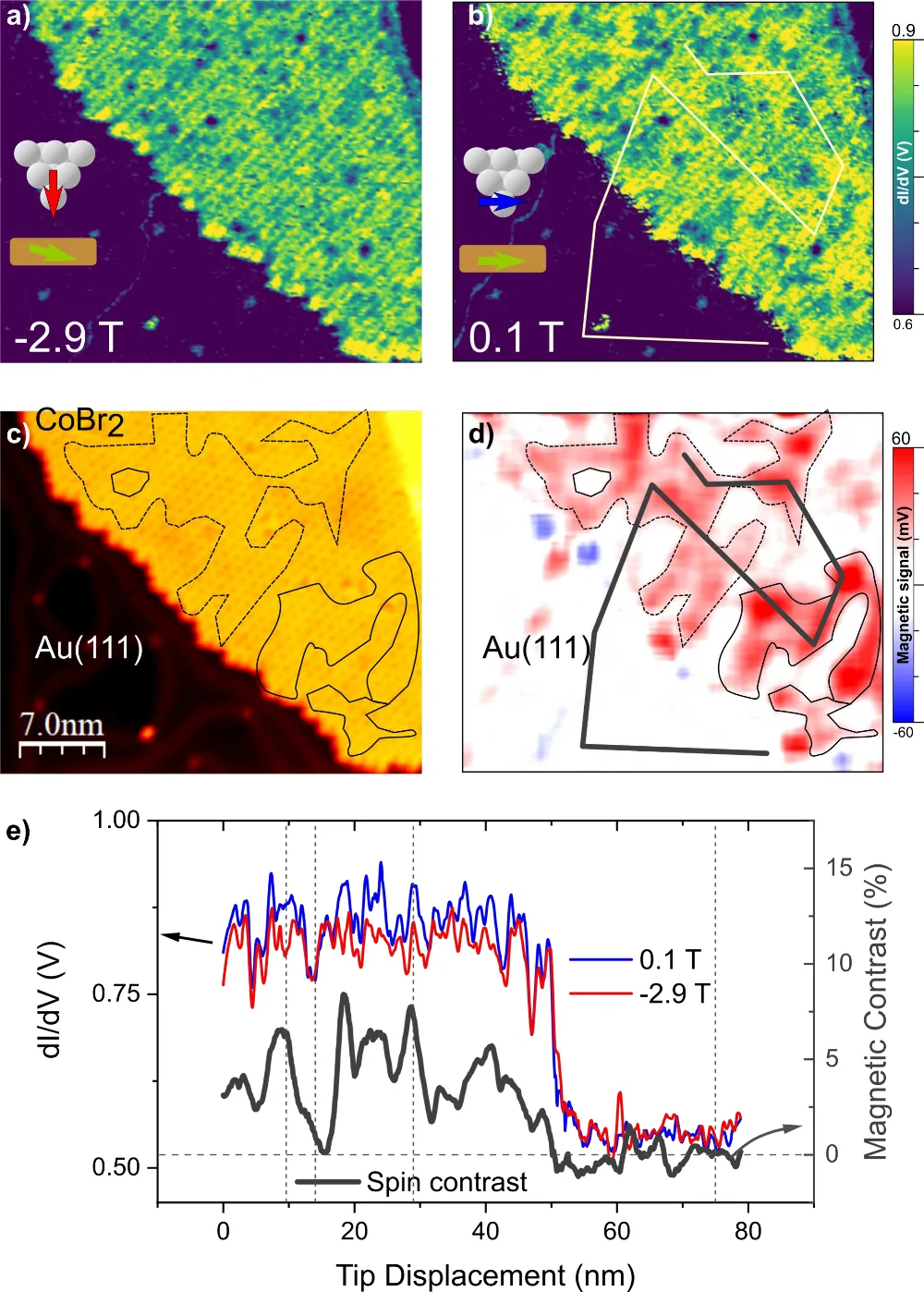
(a, b) Constant-current d*I*/d*V* maps at −2.9 and 0.1 T, respectively (set point *V* = 1.3 V and 100 pA, bias modulation 10 mV RMS). (c) Simultaneously acquired topography image of the CoBr_2_ PP phase on Au(111) during SP-STM measurements. The drawn black line in (c) shows the area without structural defects. (d) Difference between (b) and (c) showing areas where the spin-dependent tunneling conductance changes under a −2.9 T field due mainly to the reorientation of the in-plane spin-polarized Fe tip (see tip calibration in Figure S4). Signals below 2% of the total d*I*/d*V* are plotted in white. The white line in (b) and dark line in (d) represent the parts along which (e) was measured. (e) d*I*/d*V* profiles (averaged over a width of 1 nm, shown in blue and red) along the gray line profile in (d). These show an average magnetic contrast of 5% of the total conductance, which is well above the 1% uncertainty level of these measurements, as can be seen in the Au(111) part of the profile. The magnetic contrast, shown in black, is defined as the difference between the spin-dependent tunneling conductance for parallel and perpendicular tip and sample spin orientations, normalized by their average conductance: [(d*I*/d*V*)_↑↑_ – (d*I*/d*V*)_↑→_]/⟨^1^/_2_[(d*I*/d*V*)_↑↑_ + (d*I*/d*V*)_↑→_]⟩_CoBr_2__, where ⟨···⟩_CoBr_2__ denotes the mean conductance of the CoBr_2_ island.

The measurements were performed using a Fe-coated W tip with in-plane magnetic sensitivity at zero field. Under large enough applied magnetic fields, the tip magnetization rotates toward the out-of-plane direction, as confirmed by calibration measurements (Figure S3), enabling controlled variation of the spin sensitivity direction. Control experiments at equivalent magnetic states (Figure S4) further support the magnetic origin of the observed contrast. The magnetic contribution to the tunneling conductance amounts to approximately 5% of the total signal, corresponding to a maximum contrast about 4 times larger than the experimental noise level (Figure 3e).

The SP-STM measurements were repeated over the full field range from −3 to 3 T using an out-of-plane (OOP) sensitive bulk Cr tip (see tip calibration in Figure S5). Under these conditions, the field-dependent signal is negligible. This absence of contrast indicates that, within the explored field range, the magnetization of CoBr_2_ remains predominantly in-plane, suggesting a strong magnetocrystalline anisotropy. These observations are consistent with the X-ray magnetic circular dichroism (XMCD) measurements performed on surfaces predominantly covered by the PP (Figure 4), which also indicate an in-plane magnetic anisotropy.

**Figure 4:**
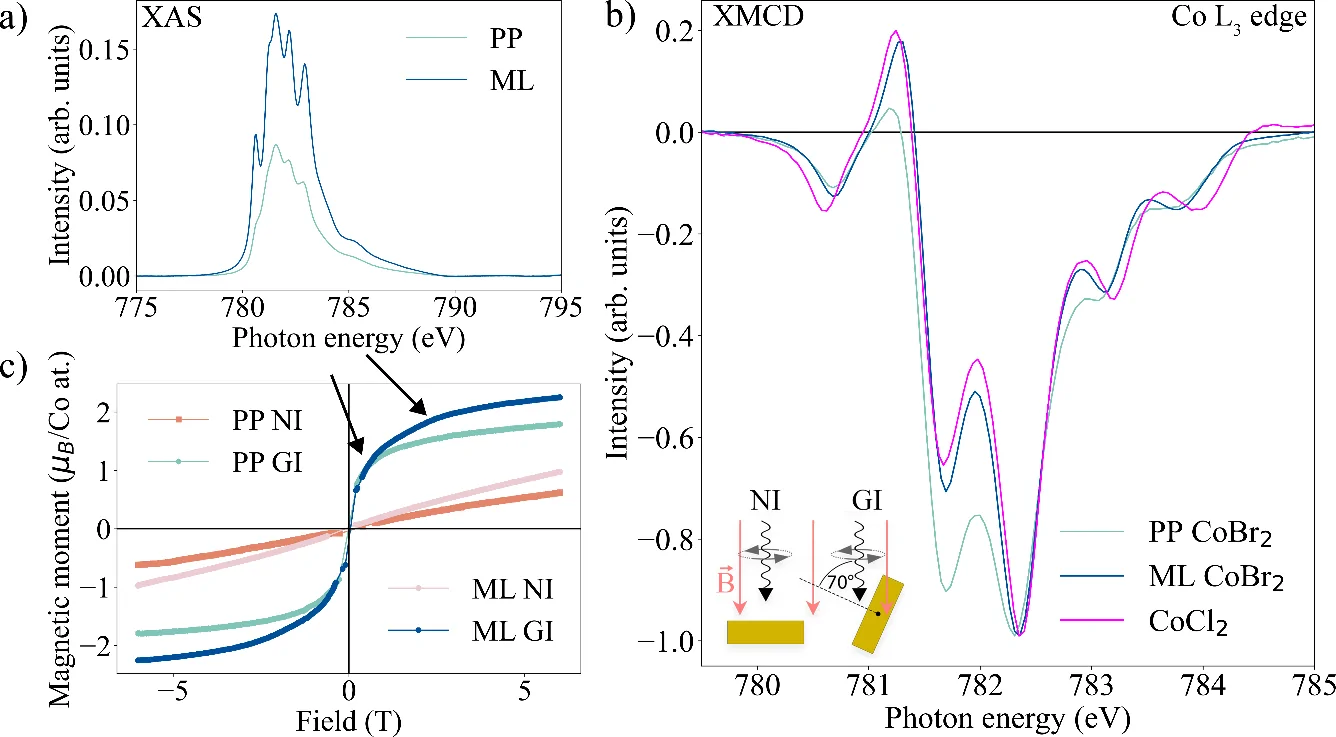
(a) XAS spectra at 2 K of the PP and ML phase of CoBr_2_ on Au(111) measured at GI and 6 T. (b) Normalized XMCD spectra at the Co L_3_ edge for bulk-like CoCl_2_ and the PP and ML phase of CoBr_2_. (c) Overlay of the NI and GI magnetization loops of the PP and ML of CoBr_2_ measured at 2 K. The magnetization loops are normalized to the sum-rule-calculated total magnetic moment. The black arrows indicate the steps inside the ML magnetization loop.

The magnetic contrast observed with the Fe-coated tip (Figure 3d,e) arises from the relative orientation between tip and sample magnetization, evolving from nearly parallel (in-plane) at low field to approximately orthogonal at high field. This behavior reflects the rotation of the tip magnetization, while the in-plane spin projection of CoBr_2_ remains dominant across the whole field range.

The resulting magnetic contrast in the SP-STM images is relatively small, pointing to a small spin polarization of the PP under external fields up to 3 T. In addition, structural defects play a key role in suppressing the local magnetic order. Both types of point defects identified in the PP, including the triangular defects observed at high bias, contribute to this suppression. This is directly evidenced in Figure 3d), where only regions free of structural defects (enclosed by the contours drawn in black) exhibit a measurable spin-polarized signal, while defect-rich areas show no detectable magnetic contrast.

The SP-STM data reveal that ferromagnetic order in the precursor phase emerges locally in structurally ordered regions and is strongly affected by point defects and disorder. However, the spatial extent of this magnetic order cannot be determined from local measurements alone. To probe its macroscopic behavior, we performed XMCD measurements, which access the average magnetization over large surface areas (μm). This complementary approach allows us to directly correlate atomic-scale magnetic contrast with long-range magnetic order in structurally distinct phases of ultrathin CoBr_2_.

To assess whether the magnetic order observed by SP-STM in the precursor phase (PP) persists at the macroscopic scale, we performed XMCD measurements at the Co L_2,3_ edge. Measurements were carried out at 2 K under UHV conditions, probing the average magnetization in samples with controlled coverage that isolate the PP and a mixed-phase preparation. Figure 4a shows the X-ray absorption spectra (XAS) of the PP (green), compared to the mixed-phase sample. Both spectra exhibit characteristic multiplet features consistent with the high-spin Co^2+^ configuration. Subtle differences in relative peak intensities, particularly in the 781–782.5 eV range, indicate changes in the local coordination environment rather than variations in oxidation state.^15^ The magnetic anisotropy was probed by varying the incidence angle of the X-ray beam from normal incidence (NI) to grazing incidence (GI) geometry under a constant magnetic field of 6 T.

Clear XMCD signals are detected in both the PP and ML samples, unambiguously demonstrating the presence of a net magnetic moment in both structural phases. The XMCD intensity strongly depends on the incidence angle of the X-ray beam, reaching its maximum at GI and vanishing at NI. This behavior indicates a predominantly in-plane magnetization for both phases, in agreement with the SP-STM results and with previous reports on bulk systems^24^ and related compounds such as CoCl_2_.

Figure 4b compares the XMCD spectra of the PP and ML phases of CoBr_2_ with those of CoCl_2_. The ML phase shows a close correspondence with CoCl_2_, consistent with a bulk-like electronic and magnetic structure. In contrast, the PP exhibits a markedly different spectral shape, particularly in the relative intensity of the L_3_ multiplet features, reflecting its distinct local coordination and electronic configuration. Minor deviations in the ML spectrum arise from the presence of residual PP contributions due to nonuniform coverage.

Magnetization loops measured at fixed temperature point to a hysteretic response in both phases. Figure 4c shows the loops acquired at 2 K for the PP and ML phases of CoBr_2_ on Au(111), under NI and GI. For the ML phase, the saturation field is around 2.5–3 T, whereas the PP saturates at lower fields (1 T) with a more gradual approach to saturation, consistent with weaker exchange interactions and in agreement with the SP-STM observations. The almost linear response in NI leads to a very small out-of-plane magnetization component at highest field of 6 T, in agreement with the relative tip–sample magnetic moments at 3 T deduced from the SP-STM measurements.

Measurements at grazing incidence confirm that both phases reach full saturation under high magnetic fields (6 T), while displaying distinct magnetization behaviors. The PP exhibits a single inflection point near 1 T, whereas samples with higher CoBr_2_ coverage show two inflection points: one around 1 T, associated with the PP, and a second near 2.5 T, corresponding to the ML. This two-step magnetization curve provides direct evidence of the coexistence of PP and ML phases, each contributing independently to the overall magnetization.

Although multistep magnetization can, in principle, arise from antiferromagnetic (AFM) coupling between layers, this scenario can be ruled out in our system. Previous studies have shown that the PP does not persist beneath the ML as a buried interface, but instead fully transforms into the bulk-like monolayer structure.^15^

Furthermore, no signatures of AFM coupling have been reported in analogous systems such as CoCl_2_/Au­(111), where the XMCD spectral shape remains unchanged across different coverages.^14^ These observations confirm that the multistep magnetization originates from the lateral coexistence of structurally and magnetically distinct surface phases.

To further assess the magnetic stability of the PP phase, we analyzed the temperature dependence of the XMCD signal and extracted the magnetic moments using sum rule analysis.^25–27^ As shown in Table 1, the magnetic moment per Co atom decreases more rapidly with increasing temperature in the PP than in the ML, indicating a reduced robustness of the magnetic order in the interfacial phase. This behavior reflects the weaker magnetic interactions in the PP, consistent with its distinct structural and electronic configuration. In particular, the reduced average moment is in agreement with the spatially inhomogeneous magnetic response observed in the SP-STM measurements (Figure 3), linking local magnetic disorder to the macroscopic magnetic properties.

**Table 1:** Samples Measured by XMCD for Different Coverages (Θ) at *B* = 6 T^a^

		μ (μ_B_/Co atom)					
		NI			GI		
Θ (ML)	*T* (K)	*m*_s,eff_	*m*_*l*_	*m*_tot_	*m*_s,eff_	*m*_*l*_	*m*_tot_
PP	2	0.43	0.20	0.63	1.42	0.37	1.79
	8	–	–	–	1.48	0.29	1.77
	13	–	–	–	1.21	0.32	1.53
	18	–	–	–	1.02	0.33	1.35
ML	2	0.75	0.22	0.97	1.76	0.49	2.25
	8	–	–	–	1.45	0.62	2.07
	13	–	–	–	1.34	0.53	1.87
	18	–	–	–	1.32	0.40	1.72

a The magnetic moments (μ) are calculated for the in-plane and out-of-plane measurements. The total magnetic moment *m*_tot_ per Co atom is the sum of the effective spin moment *m*_s,eff_ and the orbital moment *m*_*l*_. The measured moments are shown for NI and GI with an error of ±10%.

Figure 5b summarizes the critical temperatures (*T*_c_) of both phases, extracted from Arrott plot analysis of the temperature-dependent magnetization loops acquired at grazing incidence. The ML exhibits a critical temperature of approximately 14 K, while the PP orders magnetically below 12 K. Although reduced, this value suggests that the precursor phase supports spontaneous magnetization at cryogenic temperatures, despite the absence of bulk-like stacking or long-range structural order.

**Figure 5:**
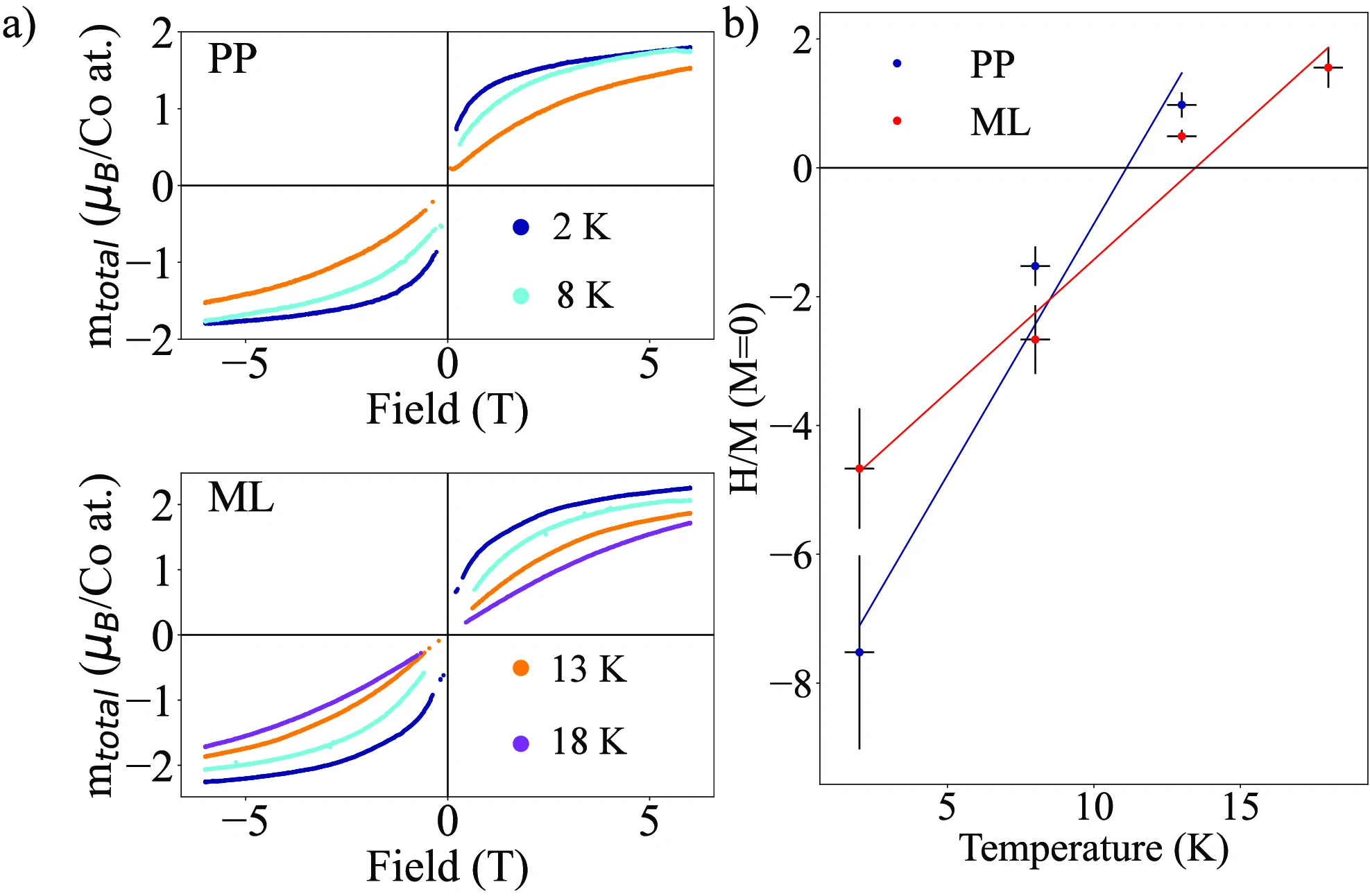
(a) Temperature-dependent magnetization loops of the PP (top) and ML (bottom) acquired at GI. The magnetization loops are normalized to the total magnetic moment values. The temperature color code given in the legend applies to both the PP and the ML loops. Each loop was recorded in both forward and backward field-sweep directions, and a single branch of the two sweeps is shown to reduce noise. This is justified since the loops pass through zero without significant coercivity and since in TEY-detected XMCD the low-field region, where coercivity could in principle be resolved, is dominated by the TEY peak. (b) Critical temperatures extracted via Arrott plot calculation.

The temperature-dependent magnetization loops (Figure 5a) show a similar overall evolution for both phases. However, in the ML phase, the step-like features observed at 2 K progressively disappear at higher temperatures, suggesting a more complex thermal evolution that may reflect additional contributions from coexisting phases.

The strong in-plane anisotropy observed in SP-STM is also reflected in the angular dependence of the XMCD magnetization loops. As shown in Figure 4c, measurements at normal incidence show no sign of saturation up to 6 T, indicating that the out-of-plane direction corresponds to the hard axis. By extrapolating the NI and GI magnetization curves to their intersection point, we estimate an anisotropy field of ∼19.9 T for the PP and ∼14.08 T for the ML. These correspond to magnetic anisotropy energies of approximately 0.8 and 0.7 meV per Co atom, respectively, based on *H*·μ_at_/2 = *E*_a_ ^28^ and assuming spin moments μ_at_ of 1.41μ_B_ (PP) and 1.76μ_B_ (ML). These values are in good agreement with theoretical predictions for Co-based monolayer systems, confirming the strong anisotropy and stability of the magnetic ground state in both phases.

Importantly, our results demonstrate that the interfacial precursor phase of CoBr_2_, despite its reduced coordination and nonbulk geometry, hosts long-range ferromagnetic order at both the atomic and macroscopic scales. This finding challenges the conventional view that robust two-dimensional ferromagnetism in transition metal dihalides is restricted to well-ordered monolayer structures. Instead, it reveals that structurally unconventional, surface-confined phases can sustain spontaneous magnetization, provided sufficient local electronic and structural coherence is preserved. A two-dimensional isotropic Heisenberg magnet cannot order at finite temperature,^29^ so magnetic anisotropy is the key ingredient for magnetic order in the 2D limit.^2^ The established 2D magnets CrI_3_, Cr_2_Ge_2_Te_6_ and Fe_3_GeTe_2_ rely on out-of-plane anisotropy,^7,8,30^ whereas the in-plane anisotropy of CoBr_2_ is closer to the 2D-XY behavior of monolayer CrCl_3_ ^31^ and to the noncollinear order of NiBr_2_ on Au(111).^13^ The key distinction is structural. These systems are bulk-like van der Waals layers, whereas the PP is a substrate-stabilized network in which the Co is directly coordinated with the Au. In contrast, the 1T ML behaves like the established TMDH systems and closely resembles CoCl_2_ (Figure 4 b).

The closest analogy for the stabilization of magnetic order without a bulk-like lattice is found in two-dimensional metal–organic frameworks on metal surfaces, where superexchange through bridging ligands and an anisotropy set by the interface sustain ferromagnetic order despite a low density of magnetic centers.^32–35^ These systems have three things in common: a structurally coherent and ordered arrangement of magnetic centers, exchange mediated by bridging ligands, and an anisotropy set by low-symmetry coordination at the interface. The PP fulfills these conditions as well, with the Br atoms taking the role of the ligands. The network is commensurate with Au(111), the Co centers couple through Co–Br–Co pathways, and XMCD indicates a significant in-plane anisotropy of approximately 0.8 meV per Co atom. This picture is consistent with SP-STM results, in which point defects locally suppress magnetic contrast, as well as with the macroscopic order observed by XMCD.

The presence of magnetic order in the precursor phase is particularly remarkable given its reduced dimensionality and the absence of multilayer coupling. While SP-STM reveals a strong sensitivity of this magnetism to atomic-scale disorder—indicating short-range exchange interactions—XMCD measurements demonstrate that spin alignment persists over macroscopic areas.

These results establish that the magnetic order in the PP is an intrinsic property of the interfacial phase, rather than a residual effect of the bulk-like monolayer. Despite exhibiting reduced Curie temperature, magnetic moment, and coercivity compared to the ML, the PP maintains a well-defined ordered/ferromagnetic state with strong in-plane anisotropy.

More broadly, our findings reveal that interfacial phases—often regarded as transient or disordered—can stabilize robust magnetic ground states. This opens new routes to engineer magnetism in low-dimensional systems beyond the monolayer limit, without requiring ideal crystallinity or multilayer stacking.

In summary, we investigated the electronic and magnetic properties of CoBr_2_/Au­(111), in which two structurally distinct phases—the interfacial precursor phase (PP) and the bulk-like monolayer (ML)—emerge during growth. By combining atomic-scale (SP-STM/STS) and macroscopic (XAS/XMCD) techniques, we establish a direct correlation between local electronic structure and magnetic order.

Our results reveal that, despite its nonbulk geometry and strong hybridization with the substrate, the PP supports long-range ferromagnetic order with pronounced in-plane anisotropy. This magnetic state is highly sensitive to atomic-scale disorder, yet persists over macroscopic length scales, demonstrating that structural coherence at the nanoscale is sufficient to stabilize collective magnetic behavior. In contrast, the ML exhibits semiconducting character and bulk-like magnetic properties, highlighting the fundamentally different electronic and magnetic nature of the two phases.

These findings demonstrate that robust magnetism in transition metal dihalides is not limited to well-ordered monolayers but can also emerge in structurally unconventional interfacial phases. Together with the tunability of defect density and phase evolution during growth, CoBr_2_/Au­(111) provides a versatile platform for engineering magnetic functionalities in low-dimensional systems.

## Supplementary Material



## Notes

Experimental methods, schematic crystal structure, wedged sample preparation, coverage calibration XAS, Arrot plot analysis, bias-dependent STM, SP-STM tip control and calibration (PDF)
