# Supplementary material for: Magnetism beyond the Monolayer: Long-Range Order in an Interfacial Phase of CoBr2
Source: Nano Lett. 2026 Oct 19;26(42):14622–9. doi: 10.1021/acs.nanolett.6c03267 (PMC13721144; doi:10.1021/acs.nanolett.6c03267)
Supplement: Supplementary file 1 [file nl6c03267_si_001.pdf]

Supplementary Information: Magnetism Beyond the Monolayer: Long-Range Order in an Interfacial Phase of $CoBr_2$

Samuel Kerschbaumer [†,‡] David Serrate [¶,§,‖] Adriana Elizabet Candia [†,‖,⊥] Andrea Aguirre [†] Danilo Longo [#] Fadi Choueikani [@] Jorge Lobo-Checa [¶,§] Pavel Jelínek [△] Maxim Ilyn [†] Celia Rogero [†] and Sebastien E. Hadjadj [*,†]

†Centro de Física de Materiales (CSIC/UPV-EHU), 20018 Donostia-San Sebastián, Spain

‡Christine Technologies Inc., Sacramento, CA 95811, EE.UU.

¶Instituto de Nanociencia y Materiales de Aragón (INMA), CSIC-Universidad de Zaragoza, 50009 Zaragoza, Spain

§Departamento de Física de la Materia Condensada, Universidad de Zaragoza, E-50009 Zaragoza, Spain

‖Laboratorio de Microscopias Avanzadas (LMA), Universidad de Zaragoza, Zaragoza E-50018 Spain

⊥Instituto de Física del Litoral, Consejo Nacional de Investigaciones Cientificas y Tecnicas, Universidad Nacional del Litoral (IFIS-Litoral,CONICET-UNL), 3000 Santa Fe, Argentina

#CIC nanoGUNE-BRTA, 20018 Donostia-San Sebastian, Spain

@Synchrotron SOLEIL, 91190 Saint-Aubin, France

△FZU - Institute of Physics of the Czech Academy of Sciences, Cukrovarnická 10, Prague 6, CZ 16200, Czech Republic

E-mail: sebastien.hadjadj@ehu.eus

Experimental and methods

Cobalt dibromide ($CoBr_2$) films (sub-monolayer to multilayer), were grown on Au(111) using a Knudsen cell evaporator with quartz crucibles (Dodecon OMBE Source[1]). The evaporation was performed under ultra high vacuum (UHV) conditions (evaporation pressure of 10^{-8} mbar to 10^{-9} mbar) at an evaporation temperature of roughly 673 K. Just as $FeBr_2$,[2] also $CoBr_2$ has shown to grow with the substrate at room temperature. This contrasts to $FeCl_2$ and $NiCl_2$[3] and $CoCl_2$[4] which were grown at slightly elevated substrate temperatures of roughly 373 K. The amount of evaporated material was estimated via a quartz microbalance, while spin-polarized scanning tunneling microscopy (SP-STM) and low-energy electron diffraction (LEED) were used as cross-reference. The purity of the anhydrous beads of $CoBr_2$ from Sigma Aldrich used in the experiment was labeled at 99.9 %[5] and the substrate cleaning process involved standard Ar^+ sputtering and annealing cycles at 720 K. UHV conditions were preserved at all time with a base pressure of 10^{-10} mbar.

The X-ray Absorption Spectroscopy (XAS) sample of $CoBr_2$ (0.8 ML and 1.7 ML) was prepared consecutively on the same sample by incrementally adjusting a shutter across the Au(111) crystal at predefined intervals. This yields a sample with different coverages grown at the same time. A schematic representation is shown in Figure S6). These samples were used for the magnetic characterization at the DEIMOS beamline at the SOLEIL synchrotron with a degree of circular polarization of 98 %.[6] The lowest achievable measurement temperature was 2 K.[7]

Low vs. High Bias STM

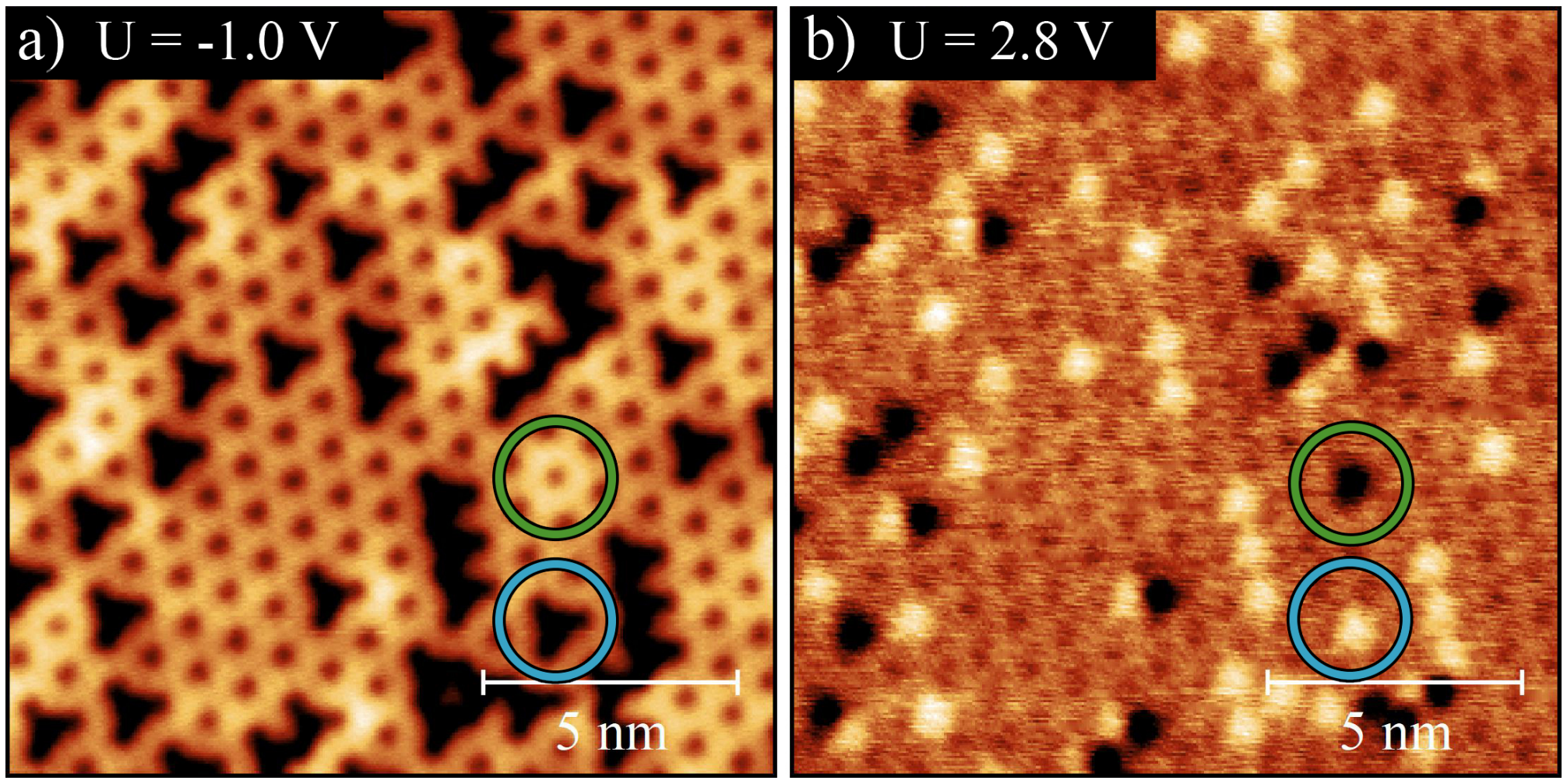

Figure S1: STS measurements on the various positions of the PP showed a significant difference of the LDOS at high positive bias. When measuring STM at the respective bias these features become visible as a change in contrast (light and dark blue circles). a) $U = -1.0$ V, $i = 10$ pA; b) $U = 2.8$ V, $i = 10$ pA.

To investigate this effect further, Figure S1 presents STM images acquired at high bias (2.8 V), which reveal two distinct types of defects. The most common are triangular defects (highlighted in light blue), appearing as bright protrusions, while a second type (green) manifests as more subtle dark depressions. Notably, areas that show sizable magnetic signal in SP-STM (Figure 4 d)), red regions) systematically coincide with zones free of both types of defects. This strongly suggests that local disorder, whether pronounced or subtle, suppresses the spin polarization in the PP phase of $CoBr_2$, disrupting magnetic ordering at the atomic scale.

STS PP vs ML

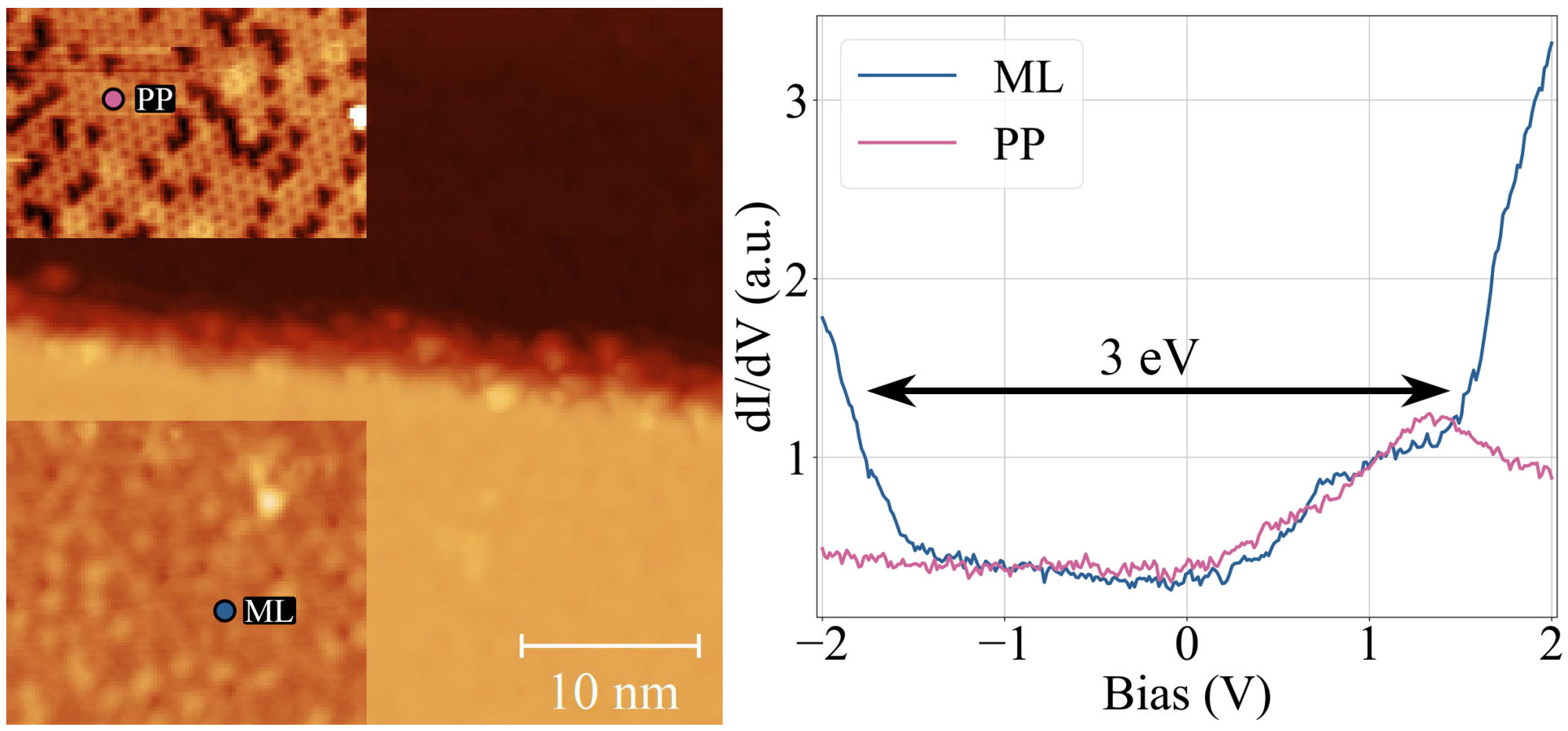

Figure S2: STM image of $CoBr_2$ on Au(111) displaying adjacent PP and ML regions separated by a step edge. The corresponding constant height STS spectra (blue and pink) reveal an apparent band gap of ∼3 eV for the ML.

SP-STM tip control and calibration

In-plane sensitive magnetic tips for spin-polarized STM (SP-STM) are prepared by depositing an 80 atomic layers thick Fe film onto a clean W-tip. The W-tip is cleaned by prior flashing at 2000 °C, which also provides a flat W surface with good crystalline quality to grow the Fe film. In this way, the Fe thin film at the tip apex is nearly parallel to the surface of the sample and acquires a pronounced in-plane magnetic shape anisotropy. Figure S3 shows the standard characterization for this kind of tip before attempting SP-STM in a material with unknown spin-resolved tunneling conductance. Since we are aiming at in-plane magnetic contrast in this case, we use as a reference the Fe monolayer (ML) on W(110), which is known to exhibit magnetic easy axis along the in-plane $[1\bar{1}0]$ direction of substrate[8,9] with huge anisotropy. For a tip with magnetic quantization axis along this direction, Fe ML stripes with opposite magnetization are imaged as regions with strong contrast in differential tunneling conductance (dI/dV), as is the case in Fig. S3 b. For this particular tip we find a spin asymmetry of 30 % at -50 mV, and approximately 20 % at higher bias above 1 V (Fig. S3 d), which is convenient for the magnetic characterization of the $CoBr_2$ feature at 1.25 V.

The marked in-plane sensitivity of the tip at zero field can be deduced from the field dependent contrast between bright and dark stripes shown in Fig. S3 c). The 3 T out-of-plane field strength is enough to overcome the shape anisotropy of the tip, leading to a tip magnetization fully perpendicular to that of the Fe stripes, and causing consequently the absence of contrast between oppositely magnetized Fe islands at large fields. As the field decreases, there is a metastable tip magnetization direction not far from in-plane at about ± 0.4 T, to finally become fully in-plane shortly after crossing zero field. The inset shows a zoom of the low field region, where a reversal of the in-plane tip magnetization component (m_x) takes place slightly below 0.1 T. m_x remains the dominant magnetization component up to about 0.2 T, after which it decreases gradually as the tip magnetization becomes out-of-plane again at higher fields. This overall behavior is characteristic of soft in-plane

ferromagnetic tips.

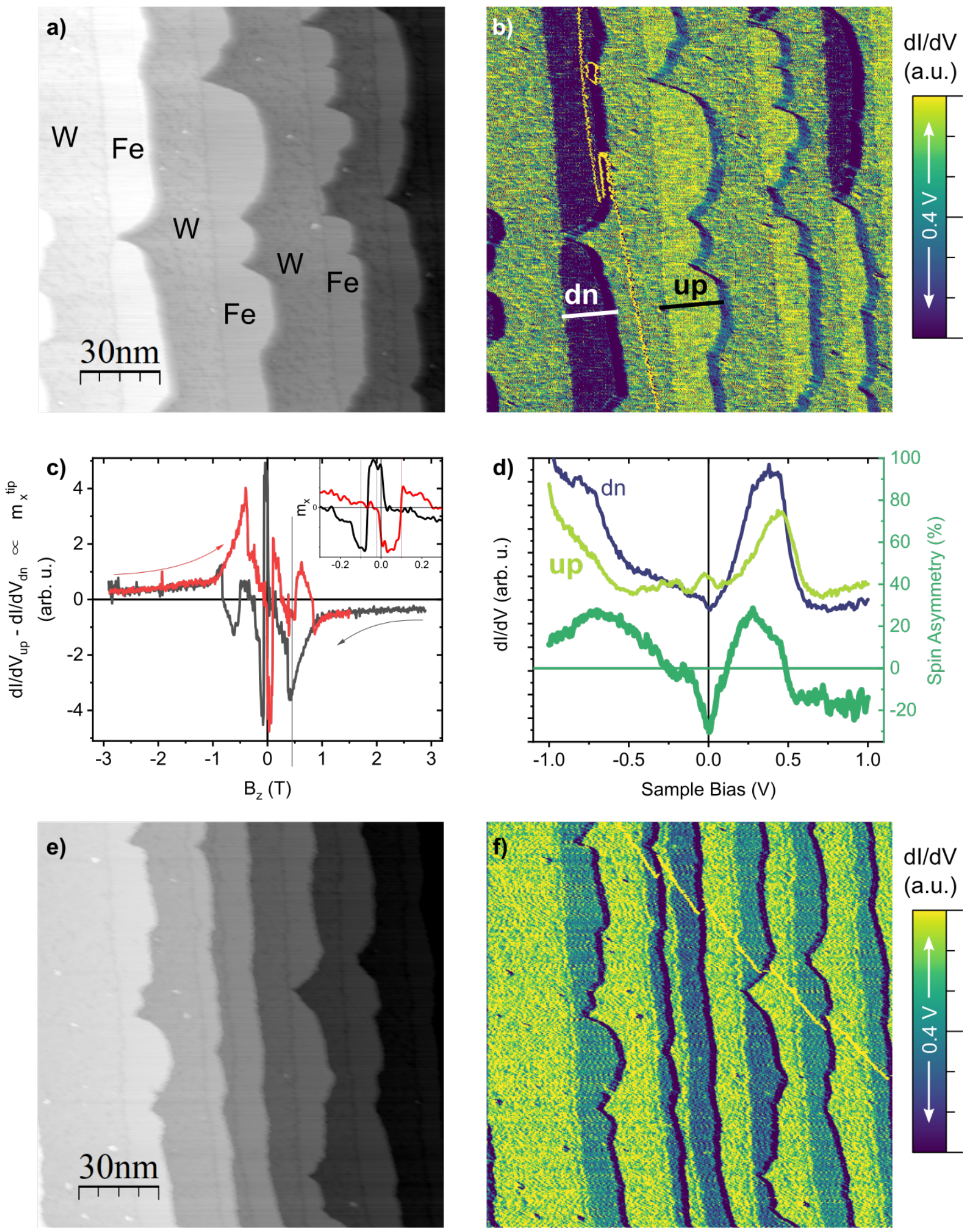

Figure S3: a),b) Simultaneous topography and dI/dV map of Fe monolayers on W(110) obtained with the Fe-coated W tip that we will use later for the $CoBr_2$ magnetic characterization in Fig. 3 of the main article. STM parameters: set point -50 mV and 50 pA, bias modulation 6 mV rms. (c) Evolution of the tip's magnetization projection onto the easy axis of the pseudomorphic Fe monolayer on W(110), which we define as OX direction. This is obtained as the difference between the averaged intensity in the black and white lines in panel (b) as the external field is driven between ±3 T. Black and red arrows denote the the field sweeping direction. (d) Spin resolved spectroscopy obtained in a bright (label 'up') and a dark (label 'dn') stripe. The spin asymmetry is calculated as $(dI/dV_{up} - dI/dV_{dn}) / (dI/dV_{up} + dI/dV_{dn})$. Stabilization set point -0.6 V and 50 pA, bias modulation 10 mV RMS. (e,f) Same as (a,b) on a fresh Fe/W(110) sample after measuring the $CoBr_2$ sample.

This very tip was then used to perform SP-STM on the $CoBr_2$ PP phase. Tip approach and sample scanning are always done at mild conditions to preserve the tip's magnetic state during the experiment. Upon completion of the $CoBr_2$ characterization, the tip magnetic contrast is investigated again with a fresh Fe/W(110) reference sample (Fig. S3 e,d). The tip obviously maintains some in-plane sensitivity thanks to a finite m_x component, although after the whole process the spin asymmetry at -50 mV has dropped to 3.5 %. This can be ascribed to subtle atomic scale changes of the tip apex which cause either a decrease of the spin polarization at that energy, or a rotation within the surface plane of the tip easy axis. In any case, this control procedure guarantees that the tip density of states is spin polarized and has in-plane sensitivity throughout the whole experiment in $CoBr_2$. Besides, the entire STM magnetic characterization process is self-consistent, as demonstrated by Figure S4 which shows the reproducibility of the $CoBr_2$ magnetic contribution to the tunneling in equivalent magnetic states. This comparison validates the magnetic nature of the contrast between magnetic states with different relative alignments of tip and sample magnetization.

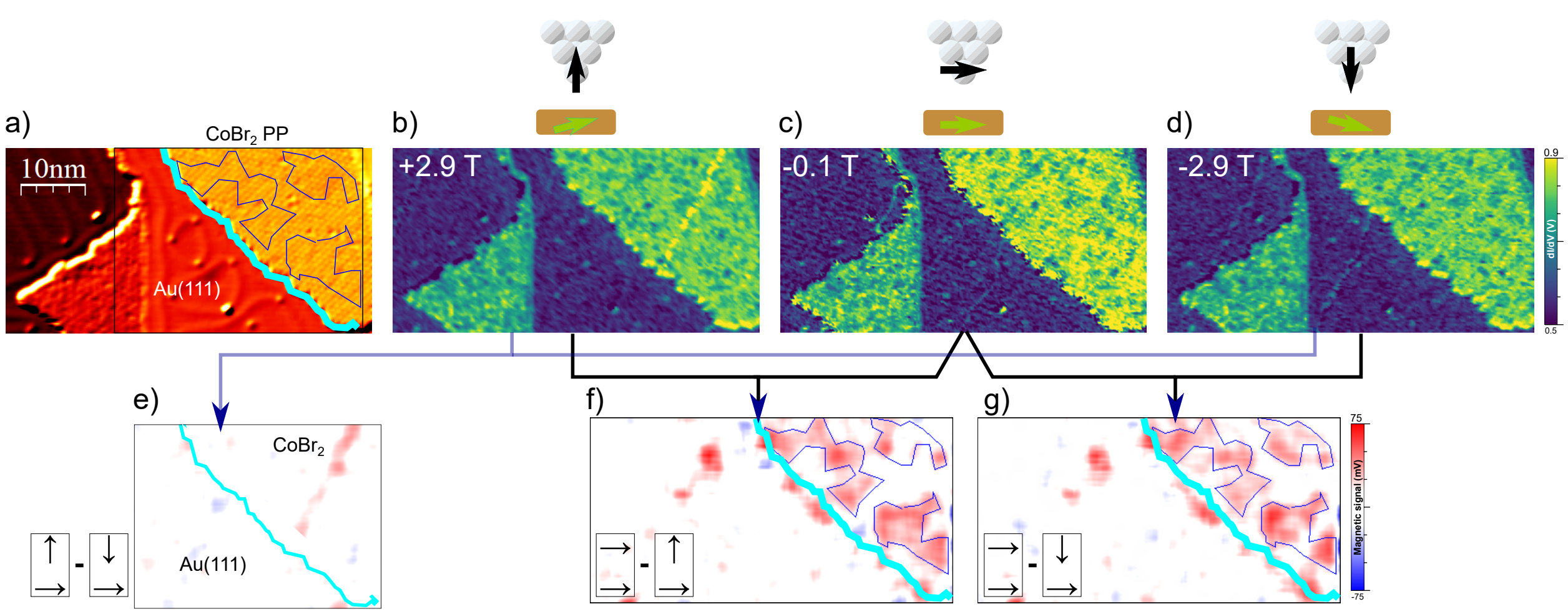

Figure S4: Control experiment of the Fe-coated W tip during SP-STM measurements of $CoBr_2$'s PP. The topography **(a)** shows two PP islands, a reconstructed with the edge highlighted in blue, and a more disordedered one. **(b,c,d)** dI/dV maps at 2.9 T, -0.1 T and -2.9 T respectively (set point $V = 1.3$ V and 100 pA, bias modulation 10 mV RMS). A sketch of the tip and sample magnetization directions is provided on top of each one. **(e)** Difference between (b)-(d) showing the absence of spin contrast in $CoBr_2$ between two equivalent alignments of tip and sample magnetization. **(f,g)** Difference between (c)-(b) and (c)-(d) respectively, showing the reproducibility of the spin contrast.

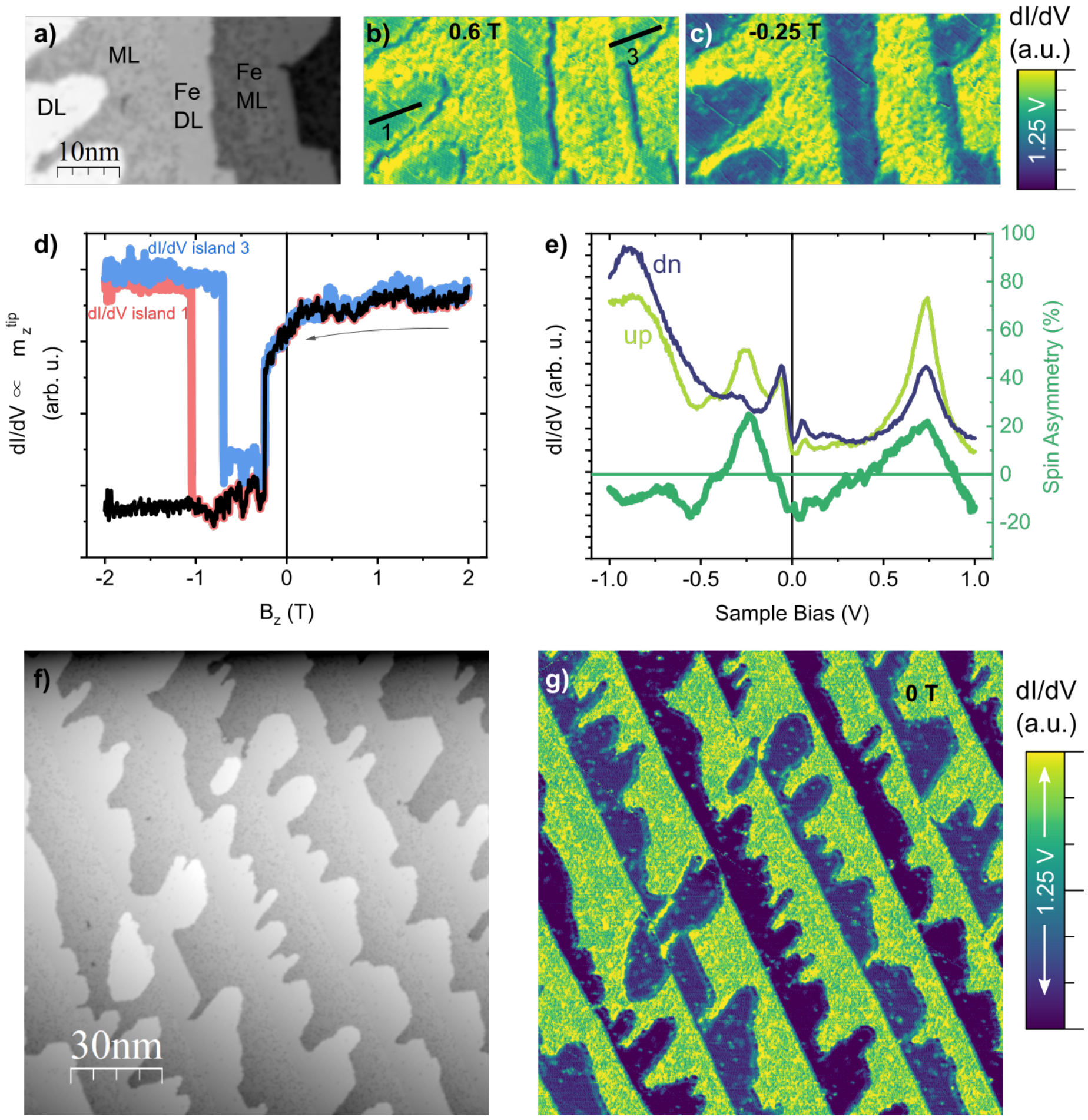

Figure S5: a),b): Simultaneous topography and dI/dV map at 0.6 T of Fe double layers (DL) on W(110) obtained with the bulk Cr tip that we will use later for the $CoBr_2$ magnetic characterization. c): Same as b) at -0.25 T after reversal of the tip magnetic moment. (d) Evolution of the tip's magnetization out-of-plane projection, which we define as OZ direction. This is obtained as dI/dV intensity of island 1 (in panels (b,c)) times the sign of the island magnetization. (e) Spin resolved spectroscopy obtained in a bright (label 'up', $B_z = 0.6$ T) and a dark (label 'dn', $B_z = -0.25$ T) DL Fe island. The spin asymmetry is calculated as $(dI/dV_{up} - dI/dV_{dn}) / (dI/dV_{up} + dI/dV_{dn})$. Stabilization set point -0.6 V and 50 pA, bias modulation 10 mV RMS. f), g): Same as a),b) at 0 T on a fresh Fe/W(110) sample after measuring the $CoBr_2$ sample. STM parameters for all images: set point -150 mV and 50 pA, bias modulation 10 mV RMS.

We also checked for spin contrast in the CoB_2 PP along the out-of-plane direction. For that purpose, out-of-plane sensitive Cr bulk tips are used. They are prepared by electrochemical etching of a pure Cr rod that is subsequently field emitted under UHV at a voltage of -120 V and 1 μA emission current. Then the tip is pulsed on the calibration sample

consisting on 1.5 ML of pseudomorphic Fe/W(110) until a contrast in dI/dV like the one shown in Figs. S5 b,c is achieved. The double layer (DL) Fe regions are known to be ferromagnetic with out-of-plane magnetic easy axis and switching fields below ~ 1 T,[10] as shown in Fig. S5 d. From the field dependent dI/dV intensity at -150 mV it is possible to infer the evolution of the out-of-plane projection of the tip magnetic moment, m_z (black curve in Fig. S5 d). The $CoBr_2$ PP was characterized with this tip at various bias voltages (including its characteristic feature at 1.3 V) and we did not detect any difference in dI/dV maps taken with opposite values of m_z (that is, above 0 T and below -0.5 T) above the experimental noise level of $\sim$1 %. This indicates that a field strength of $\pm$2 T cannot induce any measurable magnetization alignment to an out-of-plane external field. This experiment was repeated twice. After the characterization of $CoBr_2$, the bulk Cr tips typically retain their out-of-plane spin sensitivity, as shown in Figs. S5 f,g).

Wedged Sample Preparation

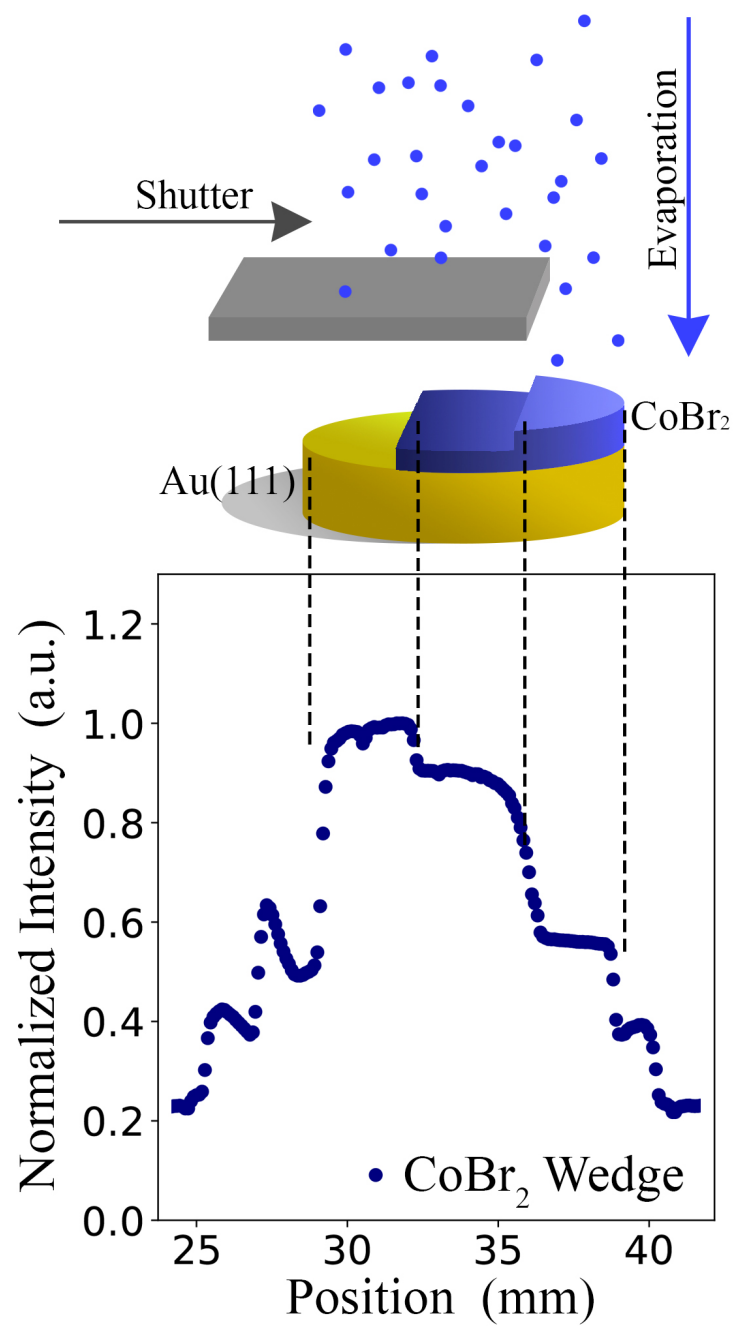

Figure S6: To prepare the wedge sample a shutter was moved in front of the sample, so that only a specific area of the crystal was exposed to the $CoBr_2$ evaporation.

Coverage Calibration XAS

The white line image of $CoBr_2$ can be used to calibrate the coverage on Au(111). Therefore, a comparison between the observed peak intensity of $FeBr_2$ on Au(111) is used.[2] Since at low temperature the Au background in the energy regime of Fe and Co behaves nearly identical,[11] the same calibration can be used. In the case of $FeBr_2$ on Au(111) a 0.7 ML sample had a average peak height of 14 %. However, since the calculation is already based on a coverage with an error we need to assume a coverage error of ≈ 0.5 ML for all XAS measurements. The coverages for the XAS measurements are thus only a rough approximation.

Sum-rule analysis of the XMCD data

The effective spin magnetic moment and orbital magnetic moment values were calculated from the areas of the L_3 and L_2 peaks of the XMCD spectra from the sum rules:[12–14]

$$m_{s,eff} = -\frac{A_{L_3} - 2 \cdot A_{L_2}}{A_{\text{Average}}} \cdot N_h \cdot \frac{1}{\sigma} \tag{1}$$

$$m_l = -\frac{2}{3} \cdot \frac{A_{L_3} + \cdot A_{L_2}}{A_{\text{Average}}} \cdot N_h \cdot \frac{1}{\sigma} \tag{2}$$

, where N_h is the number of holes (=3 for Co^{2+} in $CoCl_2$), A_{L_3}, A_{L_2} and A_{Average} are the areas of the XMCD L_3 and L_2 region and the total area of the isotropic XAS. σ represents the degree of circular polarization, which is beamline-dependent. The $m_{s,eff}$ value calculated via Equation (1) is the effective spin magnetic moment, result of the sum of the actual spin moment plus the magnetic dipole term, $\frac{7}{2}T_Z$. The background correction was performed using an asymmetrically reweighted penalized least- squares smoothing.[15]

Arrott plots analysis

In Fig. S7 the corresponding Arrott plots for the magnetization curves at different temperatures are shown. These were used to extract the critical temperature of the PP phase of $CoBr_2$ (Fig. S7 a)) and the ML phase of $CoBr_2$ (Fig. S7 b)) on Au(111).

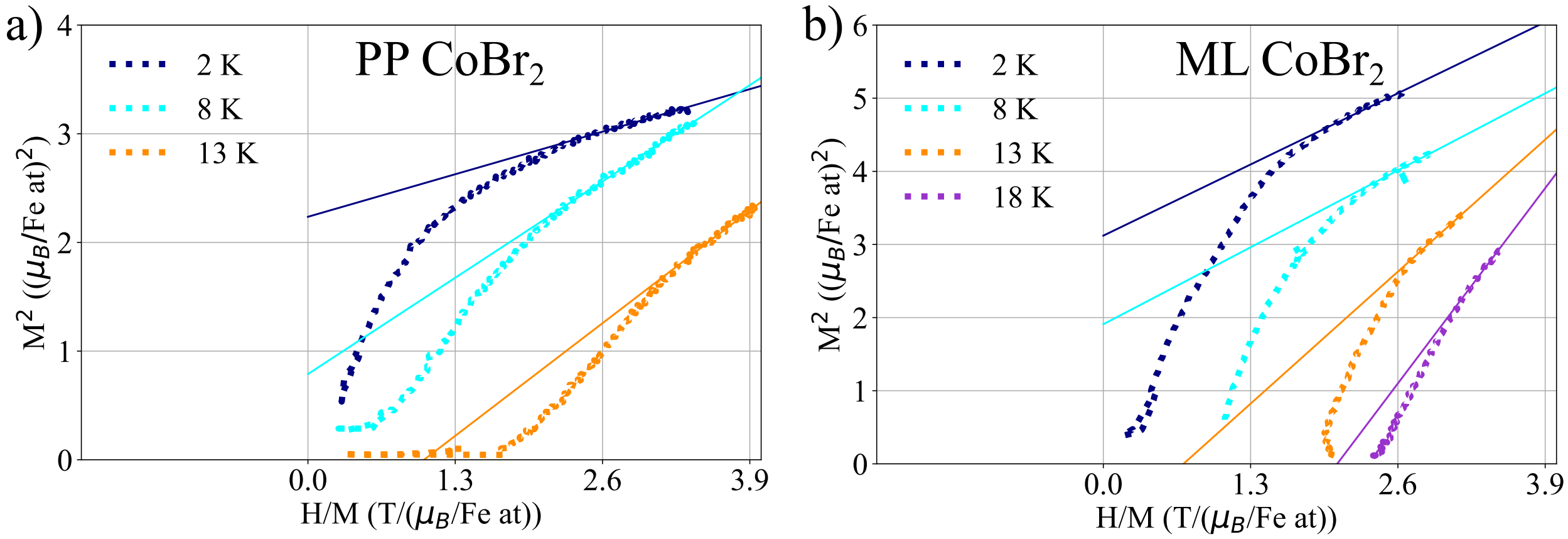

Figure S7: a) and b): Calculated Arrott plots of the temperature dependent magnetization curve measurements of the different phases of $CoBr_2$ on Au(111).

STS PP

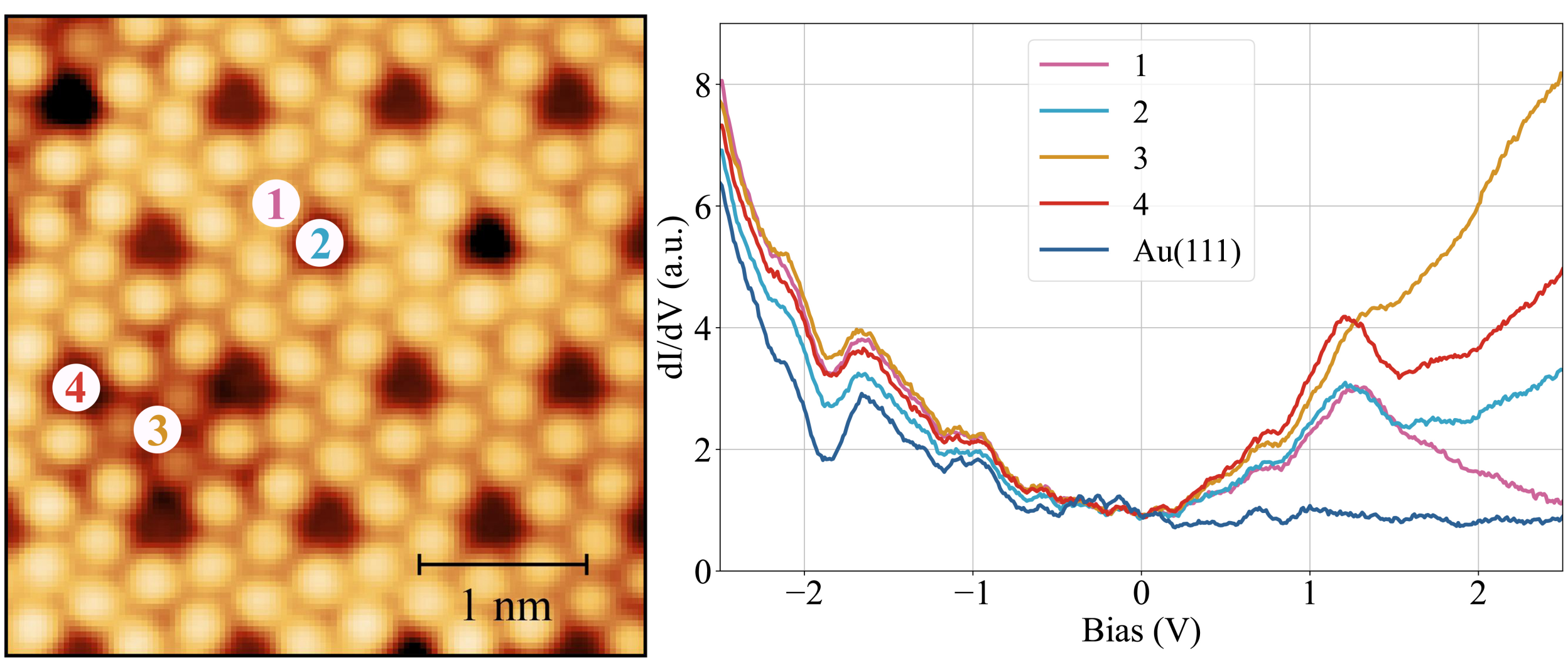

Figure S8: STS measurements of the PP of $CoBr_2$ on Au(111) reveal four distinct characteristic positions and Au(111) reference. The measured dI/dV signal remains largely constant, with notable variations only emerging beyond 1.3 V. STM: U = 5 mV, I_t = 500 pA; STS: $U = -600$ mV, I_t = 160 pA, V_{mod} = 10 mV.

References

(1) Dodecon 4x OMBE Source. `https://dodecon.de/4xOMBE.html`, Accessed: 2024-07-02.

(2) Hadjadj, S. E. et al. Epitaxial Monolayers of the Magnetic 2D Semiconductor $FeBr_2$ Grown on Au(111). Chemistry of Materials **2023**, 35, 9847–9856.

(3) Aguirre, A. et al. Ferromagnetic Order in 2D Layers of Transition Metal Dichlorides. Advanced Materials **2024**, 2402723.

(4) Kerschbaumer, S. et al. Strong In-plane Magnetic Anisotropy in Semiconducting Monolayer $CoCl_2$. ACS Nano **2025**,

(5) Aldrich, S. Cobalt(II) bromide. `https://www.sigmaaldrich.com/ES/es/product/aldrich/427136`, Accessed: 2024-07-02.

(6) Ohresser, P.; Otero, E.; Choueikani, F.; Stanescu, S.; Deschamps, F.; Ibis, L.; Moreno, T.; Polack, F.; Lagarde, B.; Marteau, F.; Scheurer, F.; Joly, L.; Kappler, J.-P.; Muller, B.; Sainctavit, P. Polarization characterization on the DEIMOS beamline using dichroism measurements. **2013**, 425, 212007.

(7) Ohresser, P. et al. DEIMOS: A beamline dedicated to dichroism measurements in the 350–2500 eV energy range. Review of Scientific Instruments **2014**, 85, 013106.

(8) Coffey, D.; Diez-Ferrer, J.; Serrate, D.; Ciria, M.; De La Fuente, C.; Arnaudas, J. Antiferromagnetic Spin Coupling between Rare Earth Adatoms and Iron Islands Probed by Spin-Polarized Tunneling. Scientific Reports **2015**, 5.

(9) Weber, N.; Wagner, K.; Elmers, H. J.; Hauschild, J.; Gradmann, U. Nanoscale spatial switching of magnetic anisotropy in pseudomorphic Fe(110) on W(110). Phys. Rev. B **1997**, 55, 14121–14124.

(10) Kubetzka, A.; Bode, M.; Pietzsch, O.; Wiesendanger, R. Spin-Polarized Scanning Tunneling Microscopy with Antiferromagnetic Probe Tips. Phys. Rev. Lett. **2002**, 88, 057201.

(11) Gold Transmission data. (2024); `https://henke.lbl.gov/cgi-bin/filter.pl`, Accessed: 2024-02-07.

(12) Kuch, W. X-ray Magnetic Circular Dichroism for Quantitative Element-Resolved Magnetic Microscopy. Physica Scripta **2004**, T109, 89.

(13) Chen, C. T.; Idzerda, Y. U.; Lin, H.-J.; Smith, N. V.; Meigs, G.; Chaban, E.; Ho, G. H.; Pellegrin, E.; Sette, F. Experimental Confirmation of the X-Ray Magnetic Circular Dichroism Sum Rules for Iron and Cobalt. Physical Review Letters **1995**, 75, 152–155.

(14) Carra, P.; Thole, B. T.; Altarelli, M.; Wang, X. X-ray circular dichroism and local magnetic fields. Physical Review Letters **1993**, 70, 694–697.

(15) Baek, S.-J.; Park, A.; Ahn, Y.-J.; Choo, J. Baseline correction using asymmetrically reweighted penalized least squares smoothing. 140, 250–257.
